# Soy Isoflavones Intake and Obesity in Chinese Adults: A Cross-Sectional Study in Shanghai, China

**DOI:** 10.3390/nu13082715

**Published:** 2021-08-06

**Authors:** Junjie Zhu, Qi Zhao, Yun Qiu, Yue Zhang, Shuheng Cui, Yuting Yu, Bo Chen, Meiying Zhu, Na Wang, Xing Liu, Yonggen Jiang, Wanghong Xu, Genming Zhao

**Affiliations:** 1 Department of Epidemiology, School of Public Health, Key Laboratory of Public Health Safety of Ministry of Education, Fudan University, Shanghai 200032, China; zhujunjie233@163.com (J.Z.); zhaoqi@shmu.edu.cn (Q.Z.); qiuyun2018@fudan.edu.cn (Y.Q.); 17111020009@fudan.edu.cn (Y.Z.); cuishuheng1995@outlook.com (S.C.); 17211020011@fudan.edu.cn (Y.Y.); chenb@fudan.edu.cn (B.C.); na.wang@fudan.edu.cn (N.W.); liuxing@fudan.edu.cn (X.L.); wanghong.xu@fudan.edu.cn (W.X.); 2 Department of Epidemiology and Health Statistics, School of Public Health, Dali University, Dali 671000, China; 3 Department of Epidemiology, School of Public Health, Shanxi Medical University, Taiyuan 030001, China; 4 Songjiang District Center for Disease Prevention and Control, Shanghai 201600, China; zmy_1963@126.com (M.Z.); Sjjkzx1106@126.com (Y.J.)

**Keywords:** soy isoflavones, body measurements, obesity, interaction, Chinese adults

## Abstract

This study was designed to examine the association of soy isoflavones (SI) intake with different body measurements indicative of obesity in Chinese adults of Shanghai, a population consuming foods rich in SI. This study used baseline data from the Shanghai Gaofeng cohort study. SI intake was measured by using a self-reported food frequency questionnaire (FFQ). A restricted cubic spline (RCS) was performed to examine the possible nonlinear relationship of SI intake with obesity. A logistic regression model was applied to estimate the odds ratios (OR) and 95% confidence interval (CI). Compared with the lowest tertile group of SI intake, the highest tertile group had a lower prevalence of obesity and central obesity. The OR for overall obesity was 0.91 (95% CI: 0.85, 0.98) in the highest versus the lowest SI tertile group; the associations differed by sex and menopausal status. A negative association was also observed between SI intake and central obesity, and a significant modifying effect of sex was found on the association. No significant interactions were observed between SI intake and physical activity (PA) levels. Our results suggest that Chinese adults with higher dietary intake of SI may be less likely to be obese, particularly for postmenopausal women.

## 1. Introduction

Obesity resulted from energy imbalance is a critical risk factor for most non-communicable diseases (NCDs), including some cancers [1,2,3]. The prevalence of obesity has been increasing globally [4]. It is estimated that about 800 million people worldwide are currently obese, which is expected result in more than USD 1 trillion of obesity-related medical costs by 2025 [5]. In Chinese adults, the prevalence of overweightness is 34.3% and the prevalence of obesity is 16.4%, and these percentages continue to increase [6]. Although the obesity-related comorbidities are complicated in etiology, a small decrease in weight or waist circumference (WC) has been associated with a significantly lower risk of obesity-related diseases [7]. Many researchers have attempted to examine the effects of increased physical activity (PA) and different nutritional strategies for weight management.

Soy isoflavones (SI) is a family of natural flavonoids, which are rich in soy and soy products. Due to its similar structure to 17 beta-estradiol, SI functions as phytoestrogens by binding to estrogen receptors and exerts weak estrogenic effects [8,9]. SI supplements have been observed to significantly decrease body weight and serum levels of lipids in animals [10,11,12,13,14,15,16]. Evidence from humans, however, is limited and inconsistent. Two cross-sectional studies reported a significant inverse association of daily consumption of dietary SI with obesity in women [17,18], but another study did not observe a significant association between total SI intake and body fat distribution [19]. Evidence from randomized controlled trials (RCTs) appeared more consistent. A recent meta-analysis of RCTs suggested that SI consumption may reduce body mass index (BMI) in women but has no effect on waist circumference [20]. Zhang et al. [21] summarized the results of nine trials (528 participants) and found that SI supplements reduced body weight in non-Asian postmenopausal women. These trials, however, were designed with a small sample size, a short time intervention, and much higher level of SI than in normal diets. It remains unclear whether the usual dietary intake of SI is associated with the risk of obesity.

Regular PA is important for maintaining a healthy weight [4]. An animal study demonstrated that combined the intervention of SI and moderate exercise could prevent body fat accumulation and increase lean body mass in ovariectomized mice [22]. In another study, no additive effect of SI and exercise was found in decreasing visceral fat mass in ovariectomized female rats [23]. Evidence in humans was mainly from intervention studies, but it was not consistent. Aubertin-Leheudre et al. [24] observed a joint effect of SI supplementation and exercise on the improvements in body weight, trunk fat mass, and BMI in Caucasian postmenopausal women. Another clinical study suggested cooperative effects of isoflavones and exercise on decreased fat mass among postmenopausal women [25]. Several other studies consistently reported that the beneficial effects on body composition pertained to resistance training or aerobic exercises but not to SI supplementation [26,27,28]. Wu et al. [29] did not find an interaction between exercise (fast walking) and capsules of isoflavones interventions on abdominal fat mass in Asian women.

Dietary SI intake is much higher in Asian populations than in their western counterparts [8]. In Chinese adults, dietary SI intake ranged from 7.8 to 25.4 mg/d, providing an opportunity to evaluate the associations of SI intake with multiple diseases in the populations [30,31,32,33]. In this study, we took advantage of the baseline data from the Shanghai Gaofeng cohort study in order to evaluate the associations between dietary intake of SI and obesity and to examine the potential modifying effects of sex and menopausal status on the associations, as well as the possible joint effect of SI intake and PA level on the risk of obesity.

## 2. Material and Methods

### 2.1. Study Design and Population

This study was based on the Shanghai Gaofeng cohort study, which is an ongoing large-scale prospective cohort study that consists of the Shanghai Suburban Adult Cohort and Biobank study (SSACB) and cohort study from Xuhui and the Minhang districts of Shanghai. The SSACB has been described in great details in our previous report [34]. Briefly, multistage cluster sampling was used for recruitment. First, 2 districts (Songjiang and Jiading) were selected according to participant willingness, health service facilities, population, geographic region, and electronic medical record system [34]. Then, 7 communities were selected as the study sites of SSACB: 4 from Songjiang (Zhongshan, Xinqiao, Sheshan, and Maogang) and 3 from Jiading (Anting, Huating, and Huangdu), based on their economic status and population. One-third of the committees or villages were randomly selected from each community. The sample designs of Xuhui and Minhang districts were similar with that of the SSACB and 5 communities were selected as the study sites: 2 from Xuhui (Tianlin and Lingyun) and 3 from Minhang (Xinzhuang, Maqiao, and Qibao). All residents in each committee who were 20 to 74 years old and lived in Shanghai for at least 5 years were eligible.

**Figure 1:**
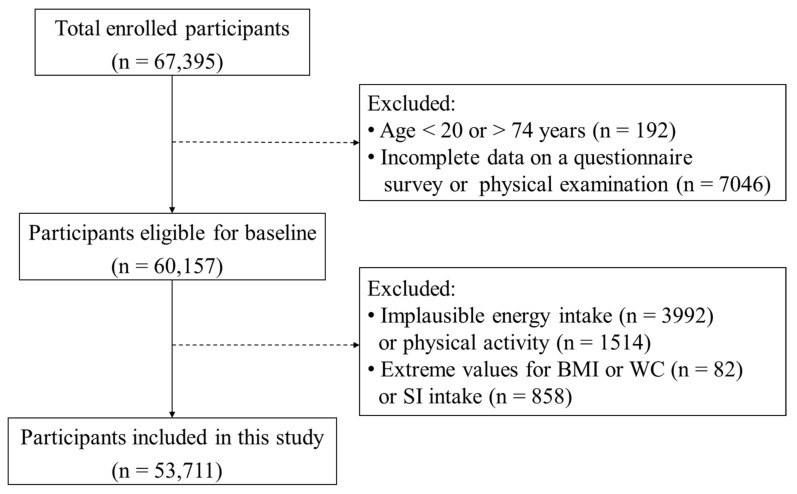
Flowchart of the study participant recruitment.

A total of 67,395 individuals were recruited and interviewed during the period of June 2016 and January 2020. Residents were excluded if they had a disability, terminal illness, perceptual impairment, or were pregnant or nursing. Individuals were also excluded if they had an incomplete questionnaire, had no physical examination results (*n* = 7046), were younger than 20 years or older than 74 years (*n* = 192), reported implausible values for energy intake (*n* = 3992) or physical activity (*n* = 1514), had extreme values of body mass index (BMI) or WC (*n* = 82), or had extreme values for the dietary intake of SI (*n* = 858). Finally, 53,711 participants were included in this analysis (Figure 1).

The participation of all individuals was approved by the Ethical Review Committee of the School of Public Health, Fudan University (IRB approval number 2016-04-0586). Written informed consent was obtained from all participants.

### 2.2. Estimated Dietary Intake of SI

Dietary intake was assessed by using a food frequency questionnaire (FFQ) including 29 food groups or items. Participants were asked how often they typically consumed each of the listed food items and what the usual serving size was during the past 12 months. Daily total energy and nutrient intakes per day were calculated on the basis of the amount of food consumed, and nutrient content was estimated with the use of the Chinese Standard Table of Food Composition [35]. The dietary intake of SI (mg/day) was calculated based on the reported consumption of soybean milk, soy milk, tofu, and other soy products (dried tofu, yuba, and fermented soy foods). Individuals with implausible total energy intake (<500 or >5000 kcal/day) and extreme values for SI intake (<1st percentile or > 99th percentile) were excluded from analysis [18,36].

### 2.3. Anthropometric Measurement and Outcome Ascertainment

Body measurements were performed three times by licensed physicians in the communities according to a standard protocol. The mean values of the three readings were recorded. Standing height (to the nearest 0.1 cm) and body weight (to the nearest 0.1 kg) were measured with the subjects standing without shoes and wearing light clothing. WC (to the nearest 0.1 cm) was measured at the midpoint between the lower rib and the upper iliac crest [34].

BMI was calculated as body weight divided by standing height squared (kg/m^2^) and classified as normal (BMI < 24 kg/m^2^), overweight (24 ≤ BMI < 28 kg/m^2^), or obesity (BMI ≥ 28 kg/m^2^) according to the criteria of weight for Chinese adults [37]; central obesity was defined as a WC of 90 cm or more in men and 85 cm or more in women. Individuals with extreme values for BMI (<15 or >40 kg/m^2^) or WC (<50 or >150 cm) were excluded from analysis [38].

### 2.4. Assessment of Covariates

Sociodemographic variables (sex, age, education level, and marital status), lifestyle factors (smoking, alcohol consumption, tea consumption, sedentary time, PA, sleep duration, and prevalent chronic diseases), and dietary data were obtained by using structured questionnaires that were administered by trained interviewers. Education levels were classified as the completion of primary school or below, middle school, high school, or college or above. Marital status was classified as married or other (unmarried, divorced, and widowed). Smoking, alcohol consumption, and tea consumption were each categorized as never or ever. PA was calculated as the metabolic equivalent of task (MET) based on the International Physical Activity Questionnaire (IPAQ) by multiplying the number of days of activity per week by the duration of the specific activity per day. PA durations over 16 h (h) per day were considered implausible [39]. Participants were divided into PA tertiles based total MET hours per week (low: <50; moderate: 51–84; high: >84). Participants were also divided into three groups based on daily sleep duration (<6 h, 6–8 h, and ≥8 h). Women were classified as premenopausal or postmenopausal (the permanent cessation of menstruation for ≥12 consecutive months). Prevalent chronic diseases considered as possible confounders were hypertension, diabetes, hyperlipidemia, chronic hepatitis, chronic kidney disease, and cancer.

### 2.5. Sensitivity Analysis

Sensitivity analysis was performed by redefining overweightness as BMI ≥ 25 kg/m^2^ and obesity as BMI ≥ 30 kg/m^2^ according to the criteria of the Center for Disease Control (CDC) [40]. Central obesity was also further defined as a WC of 80 cm or more in women [41].

### 2.6. Statistical Analysis

Participants were categorized into tertile groups according to their dietary intake of SI. Continuous variables were expressed as mean ± standard deviation (SD) or as median and interquartile range (IQR), and categorical variables were expressed as frequency (*n*) and proportion (%). The Kolmogorov–Smirnov test was used to determine whether data were normally distributed. Differences were determined by using the analysis of variance and the Wilcoxon rank-sum test for continuous variables, and the Chi-square test was used for categorical variables.

The nonlinear relationships between dietary intake of SI and body measurements were assessed by using a five-knot restricted cubic spline (RCS) at the 5th, 25th, 50th, 75th, and 95th percentiles within the logistic regression model. The reference value was the median of the lowest SI tertile. Age, sex, education level, marital status, tea consumption, sedentary time, PA level, sleep duration, energy intake, prevalent chronic diseases, menopausal status (women only), and BMI (central obesity only) were included in RCS models as covariates to control their potential confounding effects.

Multinomial or binary logistic regression models were applied to estimate odds ratios (OR) and 95% confidence intervals (CI) of SI intake with overall or central obesity. Age, sex, education level, marital status, tea consumption, sedentary time, PA level, sleep duration, energy intake, prevalent chronic diseases, menopausal status (women only), and BMI (central obesity only) were included in the models as covariates. The lowest tertile for SI intake was considered the reference group for the regression models. Tests for trends were conducted by treating the dietary intake of SI tertiles as a continuous variable in the models. Tests for interactions were conducted by adding the respective multiplicative terms in the models simultaneously. Subgroup analysis was also performed according to sex, PA level, and menopausal status.

All *p*-values were 2-tailed, and α-level of 0.05 was considered statistically significant. All analyses were performed using SAS version 9.4 (Institute Inc., Cary, NC, USA).

## 3. Results

The characteristics of the participants according to the tertiles of the dietary intake of SI are presented in Table 1. Of the participants, 20,887 were men and 32,824 were women. The dietary intake of SI ranged from 0.8 to 78.0 mg/day (median: 13.5 mg/day; IQR: 7.7, 21.4 mg/day). Compared with the participants in the lowest SI tertile group, those in the highest tertile group were younger, more likely to be men, possessed higher education, married, slept 6 to 8 h/day, possessed higher levels of PA, had higher calories, and were postmenopausal (all *p* < 0.001). However, the highest tertile was less likely to consume tea and less likely to possess chronic diseases (both *p* < 0.001).

**Figure 2:**
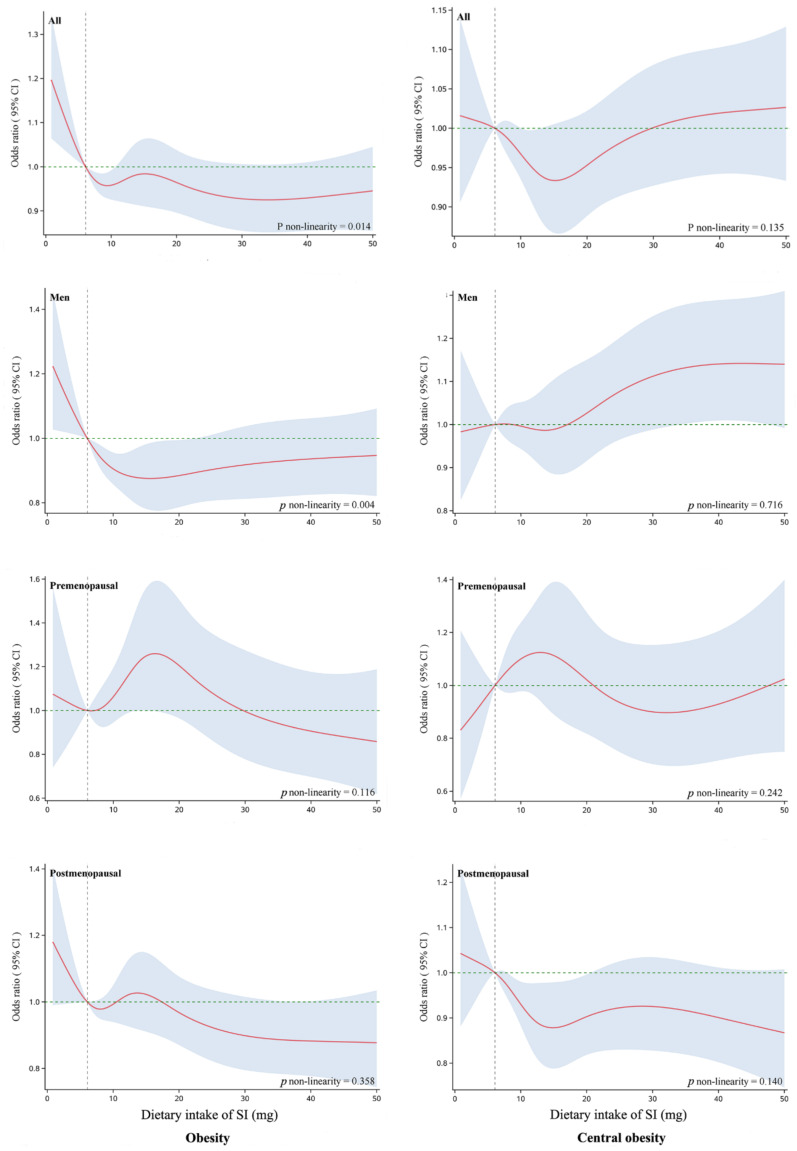
Nonlinear relationship of SI intake with obesity (left) and central obesity (right) among all subjects, men, premenopausal, and postmenopausal women. Obesity defined as BMI ≥ 28 kg/m^2^ and Central obesity as WC ≥ 90 cm for men or ≥85 cm for women. Dietary intake of SI was coded by using the RCS function with five knots arbitrarily located at the 5th, 25th, 50th, 75th, and 95th percentiles. Adjusted for sex, age, marital status, education level, alcohol drinking, smoking, tea consumption, energy intake, sedentary time, PA level, and BMI. *Y*-axis represents the adjusted odds ratio for prevalent overall obesity for any value of SI intake compared to individuals with 6.07 mg/day (the median of the first tertile of SI intake for all participants). The red solid represents the odds ratio, and the shaded area represents the 95% confidence intervals. The green horizontal short dash line represents reference line y = 1. The black vertical short dash line represents reference line χ = 6.07 mg/day. Abbreviations: SI, soy isoflavones; PA, physical activity.

We examined the possible nonlinear relationship of SI intake with obesity and central obesity in all subjects, men, premenopausal women, and postmenopausal women. As shown in Figure 2, an L-shaped relationship was observed between SI intake and obesity in men (*p* for non-linearity = 0.004). The risk of obesity decreased with increasing SI intake and then remained stable with increasing SI intake. No significant nonlinear relationship was observed for SI intake with the risk of obesity and central obesity in premenopausal and postmenopausal women (*p* for non-linearity > 0.05), but negative associations were observed in both groups.

Presented in Table 2 are body measurements by tertile intake of dietary SI. The average levels of BMI were observed to decrease with increasing tertile intake of dietary SI (*p* for trend < 0.001), while the average level of WC did not differ significantly by tertile group of SI. The prevalence of overweight/obesity and central obesity decreased with increasing tertiles (*p* for trend < 0.001), with the highest tertile group having a lower prevalence of obesity (12.0% vs. 13.4%, *p* < 0.001) and central obesity (29.3% vs. 31.1%, *p* < 0.001) relative to the lowest group.

The OR for overall obesity was 0.91 (95% CI: 0.85, 0.98) in the highest versus the lowest SI tertile group (Table 3). The associations differed by sex (*p* for interaction < 0.05), with the OR (95%CI) for the highest versus the lowest tertile groups being 0.89 (0.79, 0.99) in men and 0.91 (0.82, 1.00) in women. The negative association was observed in postmenopausal women (OR: 0.86; 95% CI: 0.77, 0.96) but not in premenopausal women (OR: 1.12; 95%CI: 0.90, 1.39).

We performed the same analyses to assess the relationship of SI intake with central obesity (Table 4). Compared with the lowest SI tertile, the highest SI tertile had no decreased risk of central obesity (OR: 0.99; 95% CI: 0.93, 1.06). However, a significant interaction of SI with sex was observed in the risk of central obesity (*p* for interaction < 0.001). There was also a significant negative association of SI intake with central obesity in women (OR: 0.91, 95% CI: 0.83, 0.99), particularly in postmenopausal women (OR: 0.90, 95% CI: 0.81, 0.99).

Sensitivity analysis observed similar results. The OR (95%CI) of overall obesity for the highest versus the lowest tertile groups was 0.80 (0.67, 0.93) in men, 1.04 (0.75, 1.43) in premenopausal women, and 0.84 (0.71, 0.98) in postmenopausal women, whereas those for central obesity were 1.01 (0.85, 1.19) and 0.95 (0.86, 1.04) for premenopausal and postmenopausal women, respectively (data not shown in the tables).

We further examined the possible joint associations of SI intake with PA level on obesity and central obesity in men, premenopausal women, and postmenopausal women. No significant interaction was observed in the three subgroups (data not shown in the tables).

## 4. Discussion

The major finding of the study is the significant inverse association of dietary intake of SI with obesity. In particular, we observed an L-shaped relationship of SI intake with overall obesity in men; we also found a significant negative association of SI intake with overall obesity and central obesity in women. We observed a significant modifying effect of sex and menopausal status on the SI-obesity associations but did not observe joint effect of SI with PA.

The dietary intake of SI varies considerably among countries, geographic regions, and even within the same country. In our study, the dietary intake of SI was moderate in that it ranged from 0.8 to 78.0 mg/day with a median of 13.5 mg/day. Previous studies of Asian populations reported that mean SI intake ranged from 6.2 to 54.3 mg/day (Japan: 26.0–54.3 mg/day; China: 6.2–40.9 mg/day) [42], which is much higher than in Western countries (1–2 mg/day) [8]. The discrepant findings on the effect of SI on obesity of previous studies may partly be explained by the differences in the amount of dietary SI intake across populations, sample size, characteristics of the participants (sex, age, and prevalent diseases), and methods used to assess SI intake.

Our finding of the negative association of SI intake and obesity, particularly among postmenopausal women, is consistent with previous studies [17,20,21]. It is possible that SI possesses pleiotropic effects in the human body by altering the metabolic balance, thereby preventing or reducing obesity [1]. SI can mimic estrogens because their structure is similar to 17-β-estradiol, and they can also bind to estrogen receptors [43]. Estrogens have known anti-obesity effects in that they regulate consumption and energy expenditure and can prevent fat accumulation in adipose tissue [44]. In addition, SI, similarly to other flavonoids, can inhibit lipogenesis and increase fatty acids (FA) β-oxidation, resulting in reduced body fat deposits [45]. Obesity disrupts immune homeostasis, increases inflammatory cell infiltration, and reduces insulin sensitivity, thereby increasing insulin resistance [46]. However, SI can activate peroxisome proliferator-activated receptors (PPARs) in various tissues, and this can suppress chronic inflammation in adipose tissue, alter lipid storage and metabolism, and improve obesity-related insulin resistance [47]. Lastly, normal intestinal microbiota metabolizes SI to equol, O-desmethylangolensin (ODMA), and other compounds, and an altered intestinal microbiota is strongly associated with obesity [48]. Thus, the ability to produce equol or ODMA following SI intake may be associated with obesity in adults [1].

The amount of SI intake seemed to have a complex relationship with obesity. We identified a non-linear (L-shaped) dose–response relationship of SI intake with obesity in men and a general downward trend of SI intake with obesity and central obesity in postmenopausal women. A meta-analysis of RCTs revealed that lower levels of SI (33.3–100 mg/day) were more effective in preventing obesity [20]. Two other studies demonstrated that a SI intake of more than 25 mg/day was needed for any biological or clinical effect [49,50]. The dose-dependent effects of SI may be related to its stimulation of catecholamine synthesis. When SI interacts with estrogen receptors in the adrenal medullary cells, they stimulate catecholamine synthesis [51], and the catecholamine-stimulating effect of SI depends on the dose: A low dose stimulates catecholamine secretion, but a high dose inhibits secretion. Notably, catecholamine release is associated with increased systemic energy consumption [52].

We found that the dietary intake of SI seemed to have different effects in men and women. In particular, the protective effect of SI intake on overall obesity seemed to be stronger in men than in women, and there was only a significant negative association of SI intake with central obesity in women. By contrast, a cross-sectional study in Korea found that obesity (based on BMI and WC) was inversely associated with a higher consumption of SI in women but not in men [18]. In general, very few previous studies examined the relationship of SI intake with obesity in men, and most studies concentrated on women, particularly postmenopausal women [19,53,54]. Some studies of male animals demonstrated that SI supplementation reduced body weight, prevented obesity, and controlled lipid metabolism, including lipogenesis and lipolysis [11,55]. One possible explanation for the sex-specific effect of SI is the different proportion and distribution of body fat by sex. Men tend to have visceral obesity, which is associated with increased cardiometabolic risk; women tend to have gynoid obesity in which most of the fat is subcutaneous, which has a weaker effect on cardiometabolic risk [56,57]. However, estrogen deficiency in postmenopausal women tends to cause visceral obesity [58,59]. SI and its metabolites can bind to estrogen and androgen receptors to exert biological effects of estrogen [60]. Moreover, estrogen levels have been suggested remain unchanged in men when supplemented with SI [60] but decreased in premenopausal women [61] and increased in postmenopausal women [62]. Thus, our findings of the negative association of SI with obesity in men and postmenopausal women suggest that SI may affect the metabolisms of visceral adipose tissue partly through a sex hormone approach. In addition, men may have a more rapid metabolism and a higher excretion rate of SI than women [63,64,65]; thus, the plasma SI level tends to be higher and longer-lasting in women. Moreover, men are more likely to have unhealthy lifestyles, such as smoking and heavy alcohol consumption, and these may increase the level of oxidative stress and attenuate the antioxidant and protective effects of SI [66,67]. Therefore, postmenopausal women may benefit more from dietary intake of SI.

We further observed a significant trend of decreased risk of obesity with increased SI intake in postmenopausal women but not in premenopausal women. Our results were consistent with two previous cross-sectional studies [17,19]. A hormonal mechanism may explain the modifying effect of menopausal status. In postmenopausal women, SI may function as an estrogen supplement and reduce lipid accumulation and alter fat distribution. However, SI may function as a competitive inhibitor of estrogen and weaken or block its effects in premenopausal women [68].

Regarding the joint effect of SI with PA, the combined intervention of walking and SI intake has been suggested to significantly decrease the fat mass in postmenopausal Japanese women [25]. In this study, we did not find a significant interaction on the risk of obesity, which is consistent with the results of previous studies [26,29]. A possible explanation for the discrepancy is that the doses of SI intervention and protocols of training were well-designed and at high levels in those studies, whereas in this study, the daily PA, including all activities such as walking, cycling, sports, and household activities, was measured based on the IPAQ, while the dietary SI intake was calculated based on an FFQ, both of which were at low levels.

The major strengths of this study included the large sample size and the population with high consumptions of soy and soy products rich in SI, which enabled us to investigate the associations between usual dietary intake of SI and obesity and provide us enough statistical power to evaluate the potential modifying effect of sex, menopausal status, and PA level on the associations.

This study had several limitations. First, we used the baseline data of a cohort study and could not infer causal relationships between dietary intake of SI and obesity. Secondly, SI consumption was calculated by using a FFQ. Recall bias is inevitable and the estimated dietary intake of SI does not always reflect its bioavailability. Thirdly, we did not consider SI supplements in our analysis and, therefore, may have underestimated SI consumption and resulted in misclassification bias. Finally, we only included total energy intake in the final model but did not consider the possible confounding effect of other nutrients (i.e., simple or complex carbohydrates intake), dietary patterns, and glycemic index of the meals on the associations.

## 5. Conclusions

The negative associations of dietary intake of SI with obesity observed in our population, particularly among postmenopausal women, suggest that postmenopausal women taking higher dietary SI may be less likely to be obese. Our results provide further evidence for the dietary guidelines to recommend the consumption of SI-rich foods such as soy bean and soy products in postmenopausal women in order to prevent obesity.

## Figures and Tables

**Table 1:** Demographic and lifestyle characteristics of the study participants according to the tertiles of SI intake.

Characteristics	All Subjects	Tertiles of Dietary SI Intake			*p* Value
	(*n* = 53,711)	T1 (*n* = 17,667)	T2 (*n* = 18,309)	T3 (*n* = 17,735)	
Age (years)	57.3 ± 11.3	58.4 ± 10.3	57.6 ± 10.7	55.8 ± 12.5	<0.001
Sex					<0.001
Men	20,887 (38.9)	6307 (35.7)	7041 (38.5)	7539 (42.5)	
Women	32,824 (61.1)	11,360 (64.3)	11,268 (61.5)	10,196 (57.5)	
Education level					<0.001
Primary school or below	18,212(33.9)	7646(43.3)	6488(35.4)	4078(23.0)	
Middle school	20,552 (38.3)	6545 (37.1)	7380 (40.3)	6627 (37.4)	
High school	8863 (16.5)	2289 (12.9)	2797 (15.3)	3777 (21.3)	
College or above	6084 (11.3)	1187 (6.7)	1644 (9.0)	3253 (18.3)	
Marital status					<0.001
Married	49,046 (91.3)	16,235 (91.9)	16,970 (92.7)	15,841 (89.3)	
Others ^a^	4665 (8.7)	1432 (8.1)	1339 (7.3)	1894 (10.7)	
Smoking					0.185
Never	42,520 (79.2)	14,048 (79.5)	14,417 (78.7)	14,055 (79.2)	
Ever	11,191 (20.8)	3619 (20.5)	3892 (21.3)	3680 (20.8)	
Alcohol drinking					0.081
Never	47,368 (88.2)	15,653 (88.6)	16,137 (88.1)	15,578 (87.8)	
Ever	6343 (11.8)	2014 (11.4)	2172 (11.9)	2157 (12.2)	
Tea consumption					<0.001
Never	37,394 (69.6)	13,015 (73.7)	13,082 (71.5)	11,297 (63.7)	
Ever	16,317 (30.4)	4652 (26.3)	5227 (28.6)	6438 (36.3)	
Sleep duration (hours)					<0.001
<6	10,006 (18.6)	3310 (18.7)	3579 (19.6)	3117 (17.6)	
6–8	34,360 (64.0)	11,143 (63.1)	11,652 (63.6)	11,565 (65.2)	
≥8	9345 (17.4)	3214 (18.2)	3078 (16.8)	3053 (17.2)	
Prevalent chronic diseases ^b^					<0.001
No	28,943 (53.9)	9172 (51.9)	10,055 (54.9)	9716 (54.8)	
Yes	24,768 (46.1)	8495 (48.1)	8254 (45.1)	8019 (45.2)	
Menopausal status (women)					<0.001
Premenopausal	8237 (25.1)	2475 (21.8)	2716 (24.1)	3046 (29.9)	
Postmenopausal	24,587 (74.9)	8885 (78.2)	8552 (75.9)	7150 (70.1)	
Sedentary time (h/day)	4 (2, 5)	3 (2, 5)	4 (2, 5)	4 (2, 5)	<0.001
PA (METs-h/week)	60.8 (36.4, 84)	59.2 (35.2, 84.0)	56 (36, 84.0)	66 (39.6, 84.0)	<0.001
PA level					<0.001
Low	17,675 (32.9)	5880 (33.3)	6146 (33.6)	5649 (31.9)	
Moderate	16,178 (30.1)	5227 (29.6)	5633 (30.8)	5318 (29.9)	
High	19,858 (37.0)	6560 (37.1)	6530 (35.7)	6768 (38.2)	
Energy intake (kcal/day)	1080 (824, 1430)	880 (696, 1127)	1058 (833, 1346)	1368 (1065, 1760)	<0.001

Data presented as mean ± SD (standard deviation) or median (IQR, interquartile range) (continuous variables) or number (%) (categorical variables). ^a^ Including unmarried, divorced, or widowed. ^b^ Including hypertension, diabetes, hyperlipemia, chronic hepatitis, chronic kidney disease, or cancers. Dietary intake of SI categorized into tertile groups (T1, T2, and T3) by using the cut-off points of 9.91 and 18.08 mg/d. Physical activity categorized into tertiles (low, moderate, and high) by using the cut-off points of 50 and 84 METs-h/week. *p* values derived from the Analysis of Variance or Wilcoxon rank-sum tests (continuous variables) or Chi-square tests (categorical variables). Abbreviations: SI, soy isoflavones; h, hours; PA, physical activity; METs, metabolic equivalents.

**Table 2:** Body measurements in the study participants according to the tertiles of SI intake.

Body Measurements	Tertiles of SI Intake (mg/d) ^a^			*p* Value	*p* for Trend
	T1 (*n* = 17,677)	T2 (*n* = 18,309)	T3 (*n* = 17,735)		
BMI (kg/m^2^)	24.4 ± 3.3	24.3 ± 3.3	24.2 ± 3.3	<0.001	<0.001
WC (cm)	82.0 ± 9.3	81.9 ± 9.3	82.1 ± 9.5	0.259	0.816
Overall obesity ^b^				<0.001	<0.001
Normal	8370 (47.4)	8893 (48.6)	8768 (49.4)		
Overweight	6931 (39.2)	7160 (39.1)	6841 (38.6)		
Obesity	2366 (13.4)	2256 (12.3)	2126 (12.0)		
Central obesity ^c^				<0.001	<0.001
No	12,168 (68.9)	12,919 (70.6)	12,537 (70.7)		
Yes	5499 (31.1)	5390 (29.4)	5198 (29.3)		

Data presented as mean ± SD (continuous variables) or number (%) (categorical variables). ^a^ Dietary intake of SI categorized into tertile groups (T1, T2, and T3) by using the cut-off points of 9.91 and 18.08 mg/d. ^b^ Normal: BMI < 24 kg/m^2^; overweight: 24 ≤ BMI < 28 kg/m^2^; obesity: BMI ≥ 28 kg/m^2^. ^c^ WC ≥ 90 cm for men or ≥85 cm for women. *p* values for Analysis of Variance (continuous variables) or Chi-square test (categorical variables). *p* for trend derived from Analysis of Variance trend tests (continuous variables) or Mantel–Haenszel Chi-square tests (categorical variables). Abbreviations: SI, Soy Isoflavones; BMI, body mass index; WC, waist circumference; SD, standard deviation.

**Table 3:** Odds ratios (OR) and 95% confidence intervals (CI) for dietary intake of SI with overall obesity.

	No. of Subjects	OR (95% CI) for Dietary Intake of SI (mg/d)					*p* for Trend	*p* for Interaction
		T1 (<9.91)	T2 (9.91–18.07)		T3 (≥18.08)			
			Overweight	Obesity	Overweight	Obesity		
All subjects								
Model 1	53,711	1.00 (ref)	0.97 (0.93, 1.02)	0.90 (0.84, 0.96)	0.94 (0.90, 0.99)	0.86 (0.80, 0.92)	<0.001	
Model 2	53,711	1.00 (ref)	0.99 (0.95, 1.04)	0.93 (0.87, 1.00)	1.00 (0.95, 1.05)	0.91 (0.85, 0.98)	0.028	
Sex ^a^								<0.001
Men	20,887	1.00 (ref)	0.96 (0.89, 1.04)	0.82 (0.73, 0.91)	1.03 (0.95, 1.11)	0.89 (0.79, 0.99)	0.047	
Women ^b^	32,824	1.00 (ref)	1.01 (0.96, 1.07)	1.01 (0.93, 1.10)	0.98 (0.91, 1.04)	0.91 (0.82, 1.00)	0.190	
PA level (METs-h/week) ^a^								0.142
Low: <50	17,675	1.00 (ref)	0.98 (0.91, 1.06)	0.92 (0.82, 1.04)	1.00 (0.92, 1.10)	0.96 (0.84, 1.09)	0.729	
Moderate: 50–84	16,178	1.00 (ref)	1.03 (0.95, 1.12)	0.86 (0.76, 0.97)	1.07 (0.98, 1.18)	0.89 (0.78, 1.02)	0.017	
High: >84	19,858	1.00 (ref)	0.99 (0.91, 1.06)	1.00 (0.90, 1.12)	0.94 (0.87, 1.02)	0.88 (0.78, 1.00)	0.096	
Menopausal status (women) ^a^								0.024
Premenopausal	8237	1.00 (ref)	1.10 (0.97, 1.26)	1.25 (1.02, 1.53)	1.07 (0.94, 1.23)	1.12 (0.90, 1.39)	0.446	
Postmenopausal	24,587	1.00 (ref)	0.99 (0.93, 1.06)	0.96 (0.87, 1.06)	0.96 (0.89, 1.03)	0.86 (0.77, 0.96)	0.029	

Overall obesity: Normal, BMI < 24 kg/m^2^; overweight, 24 ≤ BMI < 28 kg/m^2^; obesity, BMI ≥ 28 kg/m^2^. Dietary intake of SI categorized into tertile groups (T1, T2, and T3) by using the cut-off points of 9.91 and 18.08 mg/d. All models were constructed by using multinomial logistic regression methods. Model 1: unadjusted; model 2: adjustment for sex, age, education level, marital status, smoking, alcohol drinking, tea consumption, sedentary time, PA level, sleep duration, energy intake, and prevalent chronic diseases. ^a^ Adjusted for the same variables as Model 2, except for a stratifying variable; ^b^ additionally adjusted for menopausal status. Tests for trends conducted by treating dietary intake of SI tertiles as a continuous variable and tests for interactions conducted by adding the respective multiplicative terms in the models simultaneously. Abbreviations: SI, soy isoflavones; PA, physical activity; METs-h/week, metabolic equivalents-hours/week.

**Table 4:** Odds ratios (OR) and 95% confidence intervals (CI) for dietary intake of SI with central obesity.

	No. of Subjects with Central Obesity (%)	OR (95% CI) for Dietary Intake of SI (mg/d)			*p* for Trend	*p* for Interaction
		T1 (<9.91)	T2 (9.91–18.07)	T3 (≥18.08)		
All subjects						
Model 1	16,087 (30.0)	1.00 (ref)	0.92 (0.88, 0.97)	0.92 (0.88, 0.96)	0.001	
Model 2	16,087 (30.0)	1.00 (ref)	0.95 (0.90, 0.99)	0.96 (0.91, 1.01)	0.124	
Model 3	16,087 (30.0)	1.00 (ref)	0.95 (0.90, 1.01)	0.99 (0.93, 1.06)	0.927	
Sex ^a^						<0.001
Men	6700 (32.1)	1.00 (ref)	1.02 (0.92, 1.12)	1.11 (1.00, 1.24)	0.043	
Women ^b^	9387 (28.6)	1.00 (ref)	0.92 (0.85, 1.00)	0.91 (0.83, 0.99)	0.026	
PA level (METs-h/week) ^a^						0.145
Low: <50	5041 (28.5)	1.00 (ref)	0.97 (0.87, 1.07)	1.10 (0.97, 1.23)	0.149	
Moderate: 50–84	4833 (29.9)	1.00 (ref)	0.91 (0.81, 1.01)	0.91 (0.80, 1.03)	0.111	
High: >84	6213 (31.3)	1.00 (ref)	0.98 (0.89, 1.08)	0.97 (0.87, 1.07)	0.508	
Menopausal status (women) ^a^						0.211
Premenopausal	1300 (15.8)	1.00 (ref)	1.07 (0.88, 1.31)	0.94 (0.76, 1.16)	0.577	
Postmenopausal	8087 (32.9)	1.00 (ref)	0.89 (0.82, 0.97)	0.90 (0.81, 0.99)	0.015	

Central obesity defined as waist circumference ≥90 cm for men or ≥85 cm for women. Dietary intake of SI categorized into tertile groups (T1, T2, and T3) using the cut-off points of 9.91 and 18.08 mg/d. All models are constructed by using a binary logistic regression method. Model 1: unadjusted; model 2: adjusted for sex, age, education level, marital status, smoking, alcohol drinking, tea consumption, sedentary time, PA level, sleep duration, energy intake, and prevalent chronic diseases; model 3: additionally adjusted for BMI. ^a^ Adjusted for the same variables as model 3, except for a stratifying variable; ^b^ additionally adjusted for menopausal status. Tests for trends conducted by treating dietary intake of SI tertiles as a continuous variable and tests for interactions conducted by adding the respective multiplicative terms in the models simultaneously. Abbreviations: SI, soy isoflavones; PA, physical activity; METs-h/week, metabolic equivalents-hours/week.

## Data Availability

The dataset used and analyzed during the current study is available from the corresponding author upon reasonable request.

## Abbreviations

SI	Soy isoflavones
FFQ	Food frequency questionnaire
BMI	Body mass index
WC	Waist circumference
RCS	Restricted cubic spline
OR	Odds ratios
CI	Confidence interval
PA	Physical activity
NCDs	Non-communicable diseases
RCTs	Randomized controlled trials
SSACB	The Shanghai Suburban Adult Cohort and Biobank study
MET	Metabolic equivalent of task
IPAQ	International Physical Activity Questionnaire
h	Hours
SD	Standard deviation
IQR	Interquartile range
FA	Fatty acids
PPARs	Peroxisome proliferator-activated receptors
ODMA	O-desmethylangolensin
